# Livedoid Vasculopathy Mimicking Rheumatoid Vasculitis in a Patient With Active Rheumatoid Arthritis

**DOI:** 10.7759/cureus.111892

**Published:** 2026-07-01

**Authors:** Maria F Ake-Zapata, Leticia Lizama-Rubio, Ricardo F Lopez-Suarez, Andreina Moreno-Roman

**Affiliations:** 1 Internal Medicine, "Elvia Carrillo Puerto" High Specialty Regional Hospital, Institute for Social Security and Services for State Workers (ISSSTE), Merida, MEX; 2 Rheumatology, "Elvia Carrillo Puerto" High Specialty Regional Hospital, Institute for Social Security and Services for State Workers (ISSSTE), Merida, MEX; 3 Internal Medicine, Hospital General Dr. Dario Fernandez Fierro, Institute for Social Security and Services for State Workers (ISSSTE), Mexico City, MEX

**Keywords:** autoimmune disease, differential diagnosis, episcleritis, histopathology, livedoid vasculopathy, rheumatoid arthritis, rheumatoid vasculitis, rituximab, skin ulcer, thrombo-occlusive disease

## Abstract

Livedoid vasculopathy (LV) is a rare thrombo-occlusive disorder of the dermal microcirculation that may clinically resemble inflammatory vasculitis. In patients with rheumatoid arthritis (RA), the development of painful lower extremity ulcers often raises concern for rheumatoid vasculitis, making histopathological evaluation essential for accurate diagnosis and treatment selection. We present a case of a 45-year-old man with an eight-year history of seropositive RA receiving methotrexate, sulfasalazine, and baricitinib who developed painful lower extremity ulcers, edema, and episcleritis in the setting of high disease activity (Disease Activity Score 28 using C-reactive protein {DAS28-CRP} of 6.23). Given the suspicion of rheumatoid vasculitis, an extensive diagnostic workup was performed. Laboratory studies demonstrated elevated inflammatory markers and negative antineutrophil cytoplasmic antibodies. Skin biopsy revealed vascular wall hyalinization, luminal obliteration, and fibrin thrombi in the absence of inflammatory infiltrates or fibrinoid necrosis, establishing the diagnosis of livedoid vasculopathy.

Treatment with systemic glucocorticoids and rituximab, administered according to the standard rheumatoid arthritis regimen, resulted in partial improvement in ocular inflammation, joint symptoms, and cutaneous ulceration. Anticoagulant therapy was not initiated because treatment escalation was primarily directed toward uncontrolled rheumatoid arthritis and severe extra-articular manifestations. Histopathological examination demonstrated vascular wall hyalinization, luminal obliteration, and fibrin thrombi in the absence of significant vascular inflammation or fibrinoid necrosis, establishing the diagnosis of livedoid vasculopathy and excluding rheumatoid vasculitis. Although initial clinical improvement was observed, recurrent ocular symptoms and persistent lower extremity ulceration developed following interruption of biologic therapy. Histopathological confirmation demonstrating thrombotic microvascular occlusion without vascular inflammation was essential for establishing the diagnosis and avoiding misclassification as rheumatoid vasculitis. This case highlights the importance of tissue diagnosis in patients with RA presenting with ulcerative cutaneous lesions and emphasizes the need to consider livedoid vasculopathy in the differential diagnosis of suspected rheumatoid vasculitis.

## Introduction

Rheumatoid arthritis (RA) is a chronic systemic autoimmune disease characterized by persistent synovial inflammation, progressive joint destruction, and a wide spectrum of extra-articular manifestations. Although advances in disease-modifying antirheumatic drugs (DMARDs) have significantly improved outcomes, extra-articular complications continue to contribute substantially to morbidity and mortality. Among these manifestations, cutaneous involvement remains clinically relevant because it may represent either disease activity or severe vascular complications requiring prompt evaluation and treatment [1-3].

Rheumatoid vasculitis is one of the most severe extra-articular manifestations of RA and typically occurs in patients with longstanding seropositive disease. Clinical manifestations are heterogeneous and may involve the skin, peripheral nerves, and internal organs. Cutaneous findings frequently include palpable purpura, digital ischemia, and lower extremity ulcerations. Consequently, the appearance of painful ulcerative lesions in a patient with active RA often raises concern for underlying vasculitic involvement and may prompt escalation of immunosuppressive therapy [3].

Livedoid vasculopathy (LV) is a rare, chronic thrombo-occlusive disorder that affects the dermal microcirculation. Unlike inflammatory vasculitis, LV results primarily from microvascular thrombosis, endothelial dysfunction, and abnormalities in coagulation and fibrinolysis. Clinically, the disease is characterized by painful recurrent ulcerations, livedo racemosa, and the subsequent development of atrophie blanche. Because these manifestations may overlap with those observed in vasculitic disorders, distinguishing LV from inflammatory vascular diseases can be challenging on clinical grounds alone [4-6].

Histopathological examination remains the cornerstone of diagnosis. Characteristic findings include vessel wall hyalinization, luminal occlusion by fibrin thrombi, endothelial proliferation, and minimal or absent inflammatory infiltrate. These features are essential for differentiating LV from vasculitic processes, as the underlying pathophysiology, therapeutic strategies, and prognosis differ substantially. Recent reports continue to emphasize the diagnostic challenges posed by LV and highlight the importance of comprehensive clinicopathological correlation and multidisciplinary evaluation in patients presenting with chronic painful lower-extremity ulcers [4-6].

Although LV has been associated with several autoimmune conditions, including systemic lupus erythematosus, antiphospholipid syndrome, and connective tissue diseases, reports in patients with RA remain uncommon. Most of the available evidence consists of isolated case reports and small case series, suggesting that the association is rare and likely underrecognized in clinical practice. This limited evidence may contribute to diagnostic delay, particularly when ulcerative lesions develop in the setting of active rheumatic disease and are initially attributed to rheumatoid vasculitis. Furthermore, the overlapping clinical manifestations of both entities may increase the risk of misclassification when histopathological confirmation is not obtained [4,5].

## Case presentation

A 45-year-old man with an eight-year history of seropositive rheumatoid arthritis (RA) presented to our institution with painful lower extremity ulcers, edema, and unilateral ocular redness. His medical history was significant for hypertension and type 2 diabetes mellitus, both recognized risk factors for endothelial dysfunction and vascular disease. At presentation, he was receiving methotrexate, sulfasalazine, and baricitinib for RA. These therapies had been prescribed prior to the onset of cutaneous manifestations and were being used in the context of persistent disease activity.

**Figure 1:**
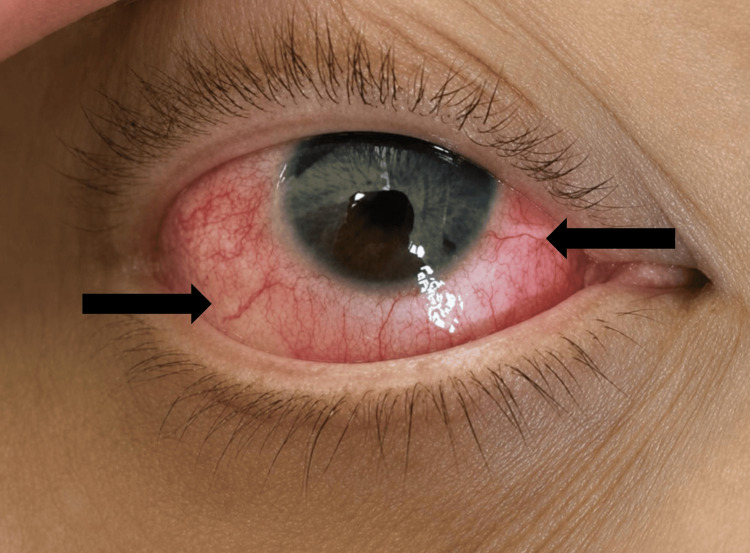
Ocular involvement associated with active disease. Representative clinical image showing unilateral ocular findings observed during the initial evaluation. Arrows highlight the most prominent areas of conjunctival vascular dilation.

Ophthalmologic examination revealed left eye episcleritis characterized by sectoral conjunctival hyperemia without corneal involvement or scleral thinning (Figure 1). Musculoskeletal evaluation demonstrated active inflammatory disease, with 17 tender and seven swollen joints, corresponding to a Disease Activity Score 28 using C-reactive protein (DAS28-CRP) of 6.23, consistent with high disease activity.

**Figure 2:**
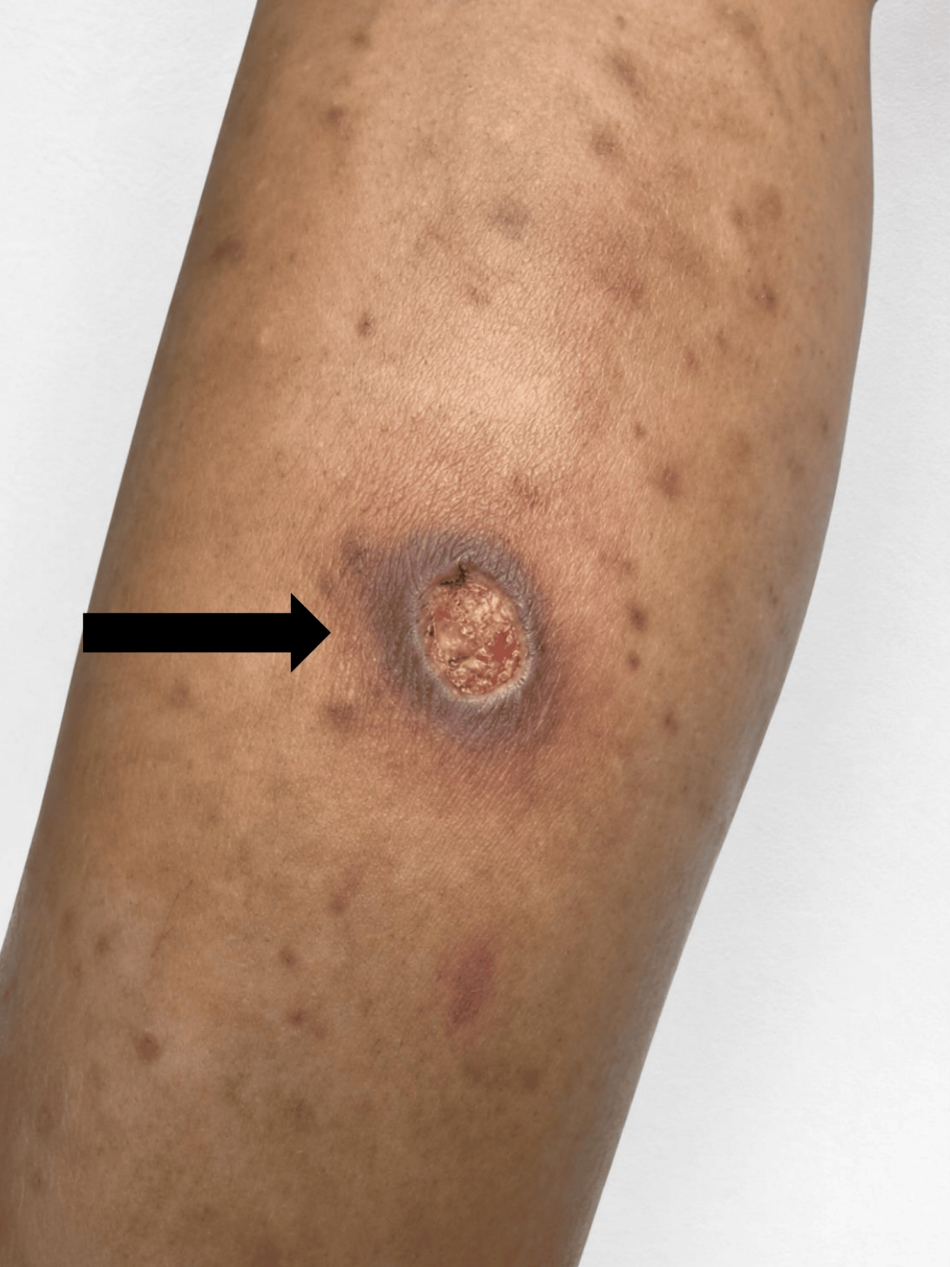
Ulcerative lesion prompting histopathological evaluation. Clinical photograph demonstrating the principal lower extremity lesion identified during the initial assessment. The arrow indicates the ulcerative focus selected for tissue sampling and diagnostic investigation.

Dermatological examination identified a solitary ulcerative lesion measuring approximately 1-1.5 cm in diameter in the posterior region of the left lower limb (sural region). The lesion exhibited well-defined borders, a fibrinous ulcer bed, and a surrounding erythematous-violaceous halo with a livedoid appearance (Figure 2). No purulent exudate, blisters, wheals, ecchymoses, plaques, or excoriations were observed. Vascular studies had reportedly been performed at an external facility prior to referral; however, the original imaging records were unavailable for review because the patient was unable to provide the corresponding study files. Given the presence of active RA, episcleritis, and ulcerative cutaneous lesions, rheumatoid vasculitis was initially considered in the differential diagnosis.

Laboratory evaluation demonstrated evidence of systemic inflammation without serological findings suggestive of ANCA-associated vasculitis. The most relevant laboratory parameters are summarized in Table 1. Elevated inflammatory markers and positive rheumatoid factor supported active rheumatoid arthritis, whereas negative C-ANCA and P-ANCA testing argued against an ANCA-associated vasculitic process.

**Table 1:** Laboratory findings at presentation.

Parameters	Results	Reference range	Interpretation
C-reactive protein (CRP)	17.5 mg/dL	<0.5 mg/dL	Elevated
Rheumatoid factor (RF)	45 IU/mL	<14 IU/mL	Positive/elevated
Procalcitonin	0.035 ng/mL	<0.05 ng/mL	Within normal limits
Urine protein	25 mg/dL	Negative	Mild proteinuria
Urine glucose	300 mg/dL	Negative	Glucosuria
Leukocyte esterase	Negative	Negative	Within normal limits
Nitrites	Negative	Negative	Within normal limits
C-ANCA	Negative	Negative	No evidence of ANCA-associated vasculitis
P-ANCA	Negative	Negative	No evidence of ANCA-associated vasculitis

Additional thrombophilia studies, including antiphospholipid antibody testing and evaluation for inherited or acquired hypercoagulable states, were not available for review. Although portions of the patient's previous diagnostic workup had reportedly been performed outside our institution, the corresponding laboratory records could not be retrieved. Consequently, the diagnosis was established based on the available clinical, laboratory, and histopathological findings.

**Figure 3:**
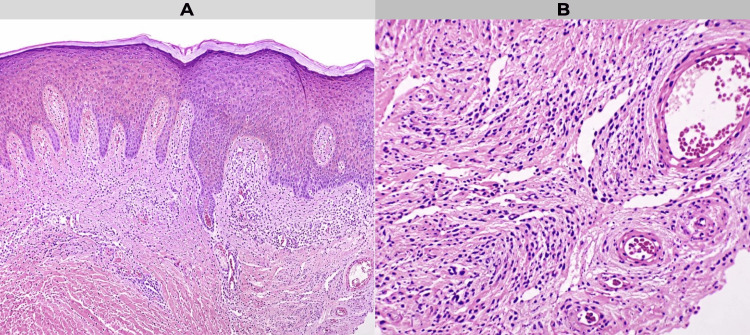
Skin biopsy demonstrating thrombotic microangiopathy. (A) Low-magnification hematoxylin and eosin section showing epidermal and dermal architectural changes associated with chronic ulceration. (B) High-magnification image demonstrating hyalinized dermal vessels with luminal occlusion by fibrin-rich thrombi and absence of significant vascular wall inflammation, supporting the diagnosis of livedoid vasculopathy.

To further characterize the lesion, an incisional biopsy was obtained from the ulcer margin. Histopathological examination demonstrated ulcerated skin with acanthosis, fibrosis, and hemosiderophages within the papillary dermis. In addition, there was proliferation of dermal blood vessels with marked hyalinization of vessel walls, luminal obliteration, and fibrin thrombi. No significant inflammatory infiltrate or fibrinoid necrosis was identified (Figures 3A, 3B). These findings were consistent with livedoid vasculopathy and effectively excluded an inflammatory vasculitic process, including rheumatoid vasculitis. Wound cultures were negative, and no evidence of dysplasia or malignancy was identified. Although vascular imaging had reportedly been performed before referral, the corresponding studies were unavailable for review because the original records could not be retrieved from the external institution.

Following the diagnostic evaluation, treatment with systemic glucocorticoids and rituximab (2 g administered every six months) was initiated in the context of active rheumatoid arthritis with severe extra-articular manifestations, including episcleritis and ulcerative cutaneous involvement. Therapeutic escalation was primarily directed toward achieving better control of systemic autoimmune disease activity rather than specifically targeting livedoid vasculopathy. Standard therapies commonly used in livedoid vasculopathy, such as anticoagulants, antiplatelet agents, pentoxifylline, or intravenous immunoglobulin, were not initiated during the period covered by the available medical records. The patient initially demonstrated clinical improvement, including partial resolution of ocular and cutaneous symptoms. However, one month later, he developed recurrent painful ocular symptoms and persistent lower extremity ulceration. At that time, joint assessment revealed 16 tender joints and no swollen joints. The recurrence was temporally associated with interruption of biologic therapy after failure to attend the subsequent rituximab administration. The patient remained under rheumatologic follow-up for ongoing management of rheumatoid arthritis and livedoid vasculopathy.

## Discussion

Rheumatoid arthritis (RA) is a chronic systemic autoimmune disease that extends beyond synovial inflammation and may involve multiple organ systems through a wide spectrum of extra-articular manifestations. Ocular, cutaneous, pulmonary, cardiovascular, and neurological complications have all been associated with persistent inflammatory activity and are generally considered markers of more severe disease. Extra-articular manifestations are particularly common in patients with longstanding seropositive RA and are associated with increased morbidity and mortality [2,3].

The present case illustrates a diagnostic challenge in a patient with active seropositive RA who developed episcleritis and a painful lower extremity ulcer. Given the coexistence of elevated inflammatory markers, positive rheumatoid factor, ocular involvement, and cutaneous ulceration, rheumatoid vasculitis represented an important initial diagnostic consideration. Rheumatoid vasculitis is a rare but severe extra-articular manifestation that typically occurs in patients with longstanding seropositive disease and uncontrolled systemic inflammation. Early recognition is essential because delayed diagnosis may result in progressive tissue injury and significant morbidity [3].

However, histopathological evaluation ultimately demonstrated findings incompatible with an inflammatory vasculitic process. An incisional biopsy obtained from the ulcer margin revealed vascular wall hyalinization, luminal obliteration, endothelial proliferation, and fibrin-rich thrombi without significant inflammatory infiltrate or fibrinoid necrosis. These findings are characteristic of livedoid vasculopathy (LV), a thrombo-occlusive microvascular disorder of the dermis rather than a primary vasculitis. The absence of vascular inflammation was particularly important in this case, as it effectively excluded rheumatoid vasculitis and supported the diagnosis of thrombotic microvascular occlusion [5,7,8].

Historically, LV was considered an inflammatory vasculitic disorder; however, current evidence supports its classification as a thrombotic vasculopathy characterized by occlusion of dermal microvessels secondary to abnormalities in coagulation, fibrinolysis, endothelial function, or blood flow. The resulting tissue ischemia leads to painful ulceration, delayed wound healing, and eventual formation of atrophie blanche. Recognition of this pathophysiological distinction is clinically relevant because diagnostic evaluation, prognosis, and therapeutic approaches differ substantially from those employed in inflammatory vasculitides [7,5].

Although LV is most commonly associated with inherited or acquired thrombophilic disorders, increasing evidence suggests that chronic systemic inflammation may contribute to endothelial dysfunction and a prothrombotic state. Persistent inflammatory activity in RA has been associated with platelet activation, increased fibrinogen production, endothelial injury, and impaired fibrinolysis, mechanisms that could theoretically favor thrombotic microvascular occlusion. Consequently, autoimmune diseases, including RA, systemic lupus erythematosus, and antiphospholipid syndrome, have been reported in association with LV [3,7,8]. However, the pathogenic relationship remains incompletely understood, and causality cannot be established from a single clinical observation. Therefore, the coexistence of active RA and LV in the present case should be interpreted as an association rather than evidence of a direct causal link.

An important aspect of this case is that the patient developed LV despite ongoing treatment with conventional and targeted disease-modifying antirheumatic drugs. This observation suggests that the presence of immunomodulatory therapy does not necessarily eliminate the risk of thrombotic cutaneous complications when residual inflammatory activity persists. At presentation, the patient exhibited high disease activity as reflected by a DAS28-CRP of 6.23, supporting the hypothesis that inadequate disease control may have contributed to the development of extra-articular manifestations and microvascular injury [2,3].

Management of LV remains challenging because high-quality evidence is limited and no universally accepted treatment algorithm exists. Current therapeutic strategies are largely directed toward the predominant pathogenic mechanism and may include anticoagulants, antiplatelet agents, pentoxifylline, intravenous immunoglobulin, vasodilators, and immunomodulatory therapies in selected patients. Direct oral anticoagulants and low-molecular-weight heparins are increasingly considered first-line options due to their favorable efficacy in thrombotic disease, whereas intravenous immunoglobulin has demonstrated promising results in refractory cases [6-8].

Although anticoagulant therapy is frequently considered a cornerstone of the management of livedoid vasculopathy, treatment decisions should be individualized based on the clinical context, associated comorbidities, and underlying systemic disease. In the present case, treatment escalation was primarily driven by uncontrolled rheumatoid arthritis and severe extra-articular manifestations. The available medical records documented initiation of systemic glucocorticoids and rituximab; however, it was not possible to determine whether anticoagulant therapy had been formally considered, as no specific documentation regarding this decision was available for review.

Emerging evidence suggests that Janus kinase inhibitors and B-cell depletion therapy may provide benefit in selected patients with autoimmune disease-associated LV. Baricitinib has shown encouraging results in recent reports, whereas rituximab has been associated with sustained remission in refractory cases and in patients with concomitant systemic autoimmune disorders [6,8]. Interestingly, the patient described in the present report developed LV despite ongoing treatment with baricitinib. This observation may reflect the heterogeneity of LV pathogenesis and suggests that JAK inhibition alone may not be sufficient to prevent thrombotic microvascular manifestations in all patients, particularly in the setting of persistent systemic inflammatory activity. Nevertheless, no causal inferences can be drawn from a single case, and further studies are needed to clarify the role of JAK inhibitors in LV associated with autoimmune diseases.

In the present patient, treatment escalation with rituximab and systemic glucocorticoids was undertaken because of persistent RA activity and severe extra-articular manifestations. Current rheumatoid arthritis treatment guidelines emphasize the importance of achieving optimal disease control through timely therapeutic adjustment when remission or low disease activity is not achieved. Although LV itself is not primarily an inflammatory vasculitis, suppression of the underlying autoimmune process may contribute to improved clinical outcomes in patients with concomitant RA by reducing endothelial activation and systemic inflammatory burden [3,9].

It is important to distinguish therapies directed at active rheumatoid arthritis from those specifically used for livedoid vasculopathy. Standard LV management frequently includes anticoagulants, antiplatelet agents, pentoxifylline, or intravenous immunoglobulin, with treatment selection guided by the presumed thrombotic and microvascular mechanisms of disease [7,8,10]. In contrast, the therapeutic strategy documented in this case was primarily aimed at controlling active RA and its extra-articular manifestations. Although anticoagulant therapy is commonly considered in LV, the available medical records did not contain sufficient information to determine whether this option had been formally evaluated during clinical decision-making.

This case highlights the critical role of histopathological confirmation in patients with rheumatoid arthritis who develop ulcerative cutaneous lesions. While rheumatoid vasculitis should remain a major diagnostic consideration in the presence of active disease and extra-articular manifestations, livedoid vasculopathy represents an important differential diagnosis that may closely mimic vasculitic disease. Accurate recognition of its thrombotic rather than inflammatory nature has direct implications for diagnostic evaluation, therapeutic decision-making, and long-term patient outcomes [7,8,10,11].

## Conclusions

Livedoid vasculopathy should be considered in the differential diagnosis of ulcerative cutaneous lesions in patients with active rheumatoid arthritis, particularly when extra-articular manifestations raise suspicion for rheumatoid vasculitis. In the present case, histopathological examination was pivotal in establishing the diagnosis by demonstrating a thrombo-occlusive microvasculopathy in the absence of significant vascular inflammation.

This report underscores the critical role of skin biopsy in differentiating vasculopathy from vasculitis, as this distinction has direct implications for diagnostic evaluation, therapeutic decision-making, and prognosis. Furthermore, this case highlights the potential contribution of persistent systemic inflammatory activity to thrombotic microvascular complications and reinforces the importance of optimizing control of the underlying autoimmune disease. Early recognition and accurate histopathological characterization remain essential for achieving appropriate management and favorable clinical outcomes.
